# Green Approaches for the Extraction of Banana Peel Phenolics Using Deep Eutectic Solvents

**DOI:** 10.3390/molecules29153672

**Published:** 2024-08-02

**Authors:** Ena Cegledi, Erika Dobroslavić, Sandra Pedisić, Ivan Magnabosca, Marija Zorić, Rina Pavić, Marija Šuto, Otilija Štargl, Maja Repajić, Ivona Elez Garofulić

**Affiliations:** 1 Faculty of Food Technology and Biotechnology, University of Zagreb, Pierottijeva 6, 10000 Zagreb, Croatia; erika.dobroslavic@pbf.unizg.hr (E.D.); imagnabosca@gmail.com (I.M.); marija.zoric888@gmail.com (M.Z.); r.pavic54@gmail.com (R.P.); otilija.stargl1@gmail.com (O.Š.);; 2 Centre for Food Technology and Biotechnology, University of Zagreb, Petra Kasandrića 3, 23000 Zadar, Croatia; sandra.pedisic@pbf.unizg.hr

**Keywords:** *Musa acuminata*, ultrasound-assisted extraction, microwave-assisted extraction, bioactives, antioxidant activity

## Abstract

Banana peels, comprising about 35% of the fruit’s weight, are often discarded, posing environmental and economic issues. This research focuses on recycling banana peel waste by optimizing advanced extraction techniques, specifically microwave-assisted (MAE) and ultrasound-assisted extraction (UAE), for the isolation of phenolic compounds. A choline chloride-based deep eutectic solvent (DES) with glycerol in a 1:3 ratio with a water content of 30% (*w*/*w*) was compared to 30% ethanol. Parameters, including sample-to-solvent ratio (SSR), extraction time, and temperature for MAE or amplitude for UAE, were varied. Extracts were analyzed for hydroxycinnamic acid (HCA) and flavonoid content, and antioxidant activity using FRAP and ABTS assays. DES outperformed ethanol, with HCA content ranging from 180.80 to 765.92 mg/100 g and flavonoid content from 96.70 to 531.08 mg/100 g, accompanied by higher antioxidant activity. Optimal MAE conditions with DES were an SSR of 1:50, a temperature of 60 °C, and a time of 10 min, whereas an SSR of 1:60, time of 5 min, and 75% amplitude were optimal for UAE. The polyphenolic profile of optimized extracts comprised 19 individual compounds belonging to the class of flavonols, flavan-3-ols, and phenolic acids. This study concluded that DESs, with their superior extraction efficiency and environmental benefits, are promising solvents for the extraction of high-value bioactive compounds from banana peels and offer significant potential for the food and pharmaceutical industries.

## 1. Introduction

Banana is one of the most popular types of fruit worldwide, as it can provide many nutrients in the human diet. It belongs to the *Musaceae* family and is divided into three genera: Musa, Ensete, and Musella, within which there are various species [1]. Bananas are usually eaten fresh or processed into flour, dried, or in the form of crisps. The annual production is over 135 million tons [2]. The banana peel is considered a by-product after processing and makes up about 35% of the weight of the fruit. Most of the peel is thrown away as waste, leading to environmental problems and economic losses [3,4]. Banana peels are traditionally used to treat various inflammations, diabetes, burns, diarrhea, and other health problems. The banana peel is known to contain numerous compounds, such as dietary fiber and phenolic compounds, which are present in greater quantities in the banana peel than in the peel of other fruits. It also has a high antioxidant activity, has an antibacterial effect, and inhibits the development of cancer cells [3,5,6]. It is, therefore, important to properly utilize banana peel as a source of numerous bioactive molecules for use in the food and pharmaceutical industries. To efficiently isolate bioactive components from plant material, advanced extraction techniques such as microwave-assisted extraction (MAE) and ultrasound-assisted extraction (UAE) are increasingly being used today. The advantages of these “green” extraction techniques compared to conventional techniques are a shorter extraction time, the use of a lower amount of solvents, no harmful effects on the environment, high efficiency, and high quality of the extracts [7].

The use of alternative environmentally friendly solvents, such as deep eutectic solvents (DESs), is also becoming increasingly popular compared to conventional solvents, which are often associated with toxicity. DESs are a new class of liquids that usually consist of two components: hydrogen-bond donors (alcohol, sugar) and hydrogen-bond acceptors (quaternary ammonium salts). These components are readily available, cheap, easy to recycle, and non-toxic, thus allowing extracts to be utilized further without eliminating the solvent [8]. DESs have a lower melting point than each individual component and are applicable to a wide range of different compounds as they have unique physicochemical properties that can be adjusted depending on the molecules to be isolated. Thus, DESs have adjustable viscosity, a wide polarity range, low volatility, low toxicity, and are non-flammable [9,10]. It has also been shown that certain DESs have a high ability to stabilize phenolic compounds and enhance the antioxidant activity of plant extracts [11]. The most extensively researched type of DES for isolating bioactive compounds is based on choline chloride, which has a strong ability to form hydrogen bonds due to the presence of its chloride ion, which is a potent hydrogen bond acceptor. This allows it to interact effectively with hydrogen-bond donors in the DES, such as organic acids, sugars, or glycerol, forming a stable eutectic mixture. Polyphenols have multiple hydroxyl groups that can form hydrogen bonds. The hydrogen-bond network in DES, facilitated by choline chloride, enhances the solubility of polyphenols by effectively disrupting intermolecular hydrogen bonds within the polyphenols and increasing their interaction with the solvent [12]. Therefore, the ability of choline chloride to act as a strong hydrogen-bond acceptor and form stable, efficient, and environmentally friendly DES makes it particularly effective for the isolation of polyphenols. All these properties make DESs excellent solvents for sustainable and environmentally friendly extraction.

As far as the authors are aware, there is no study dealing with the impact of advanced extraction techniques in combination with DES for the isolation of phenolic components from banana peels. Therefore, the aim of this study was to optimize MAE and UAE parameters for the efficient isolation of hydroxycinnamic acids (HCA) and flavonoids from banana peel extracts and to determine the antioxidant activity of the extracts obtained using the FRAP and ABTS methods. The solvent used was a DES comprising a mixture of choline chloride and glycerol in ratio 1:3 with a water content of 30% and 30% ethanol for comparison. The parameters varied were the sample:solvent ratio (g/mL) (SSR), time, and temperature for MAE, while for UAE, the amplitude was varied instead of temperature. For the optimal extracts, the phenolic composition was determined by Ultra-Performance Liquid Chromatography-Tandem Mass Spectrometry (UPLC-MS^2^).

## 2. Results and Discussion

This study aimed to investigate the influence of MAE and UAE parameters (SSR, extraction, and temperature in the case of MAE or wave amplitude in the case of UAE) on the phenolic content and antioxidant activity of banana peel extracts comparing the efficacy of DES (choline chloride:glycerol 1:3) and 30% ethanol as the extraction solvent. This DES was chosen based on a preliminary study in which the influence of various choline chloride-based DES on the HCA and flavonoid content was investigated (Table S1). The results for each specific parameter in both extraction techniques are shown in Supplementary Data (Table S2), while the results of statistical analysis are provided in Table 1 and Table 2. The content of HCA varied from 180.80 to 765.92 mg/100 g in DES and from 18.40 to 146.74 mg/100 g in 30% ethanol extracts. The content of flavonoids varied from 96.70 to 531.08 mg/100 g in DES and 64.12 to 258.18 mg/100 g in 30% ethanol, which is in large part lower than the values achieved during enzyme-assisted extraction from banana peel, where the range varied from 3.92 to 14.30 mg/g, depending on the applied extraction conditions [13]. Antioxidant activity determined by FRAP and ABTS varied from 9.23 to 70.33 mmol/100 g and 10.52 to 32.93 mmol/100 g in DESs, respectively, and 1.97 to 14.58 mmol/100 g and 1.21 to 9.12 mmol/100 g in 30% ethanol, respectively. FRAP values determined in both DES and 30% ethanol extracts were mostly higher than those obtained previously in 8 different varieties of banana with water or organic solvents [14]. The ABTS values were in the range of those determined in banana peel extracts using mixtures of methanol, ethanol, acetone, and water as a solvent [15]. Generally, higher values of all tested parameters were observed using DESs in both techniques, compared to 30% ethanol, which is consistent with previous findings where different DESs were more efficient than water, ethanol, methanol, and their mixtures [16,17,18].

### 2.1. Optimization of MAE

In the present study, the influence of SSR, extraction time, and temperature of MAE on the content of HCA and flavonoids, as well as the antioxidant activity determined by FRAP and ABTS methods, was examined, and the results of statistical analysis are shown in Table 1.

The selection of solvent and SSR are critical factors in MAE. Effective extraction depends on the solvent’s ability to absorb microwave energy, influenced by its dielectric properties [19]. Optimal SSR in MAE has been found to often range between 1:10 and 1:20 (g/mL), with higher ratios potentially increasing diffusion gradients and extraction rates [19]. Increasing the ratio beyond 1:20, however, might not yield more polyphenols, as observed in some studies, likely due to the limited extractable content from the sample matrix. Excessive solvent volumes in MAE can also hinder solvent stirring and reduce extraction efficiency in MAE, which is why it is crucial to define the optimal ratio for each solvent and sample type [20]. In the present study, SSR significantly influenced the content of HCA, flavonoids and antioxidant activity in the case of both DES and 30% ethanol. When applying DES, the highest content of HCA and flavonoids was observed at 1:50, while further increase had no significant effect. As for antioxidant activity, the 1:40 ratio resulted in the highest values determined by FRAP, while 1:60 was optimal in ABTS. It is likely that a lower ratio of DES was sufficient to achieve high yields due to the enhanced solubility and strong hydrogen bonding interactions, which disrupt plant cell walls and solubilize polyphenols more efficiently, leading to better extraction performance even with a smaller volume of solvent [21]. Generally, the applied SSR may vary significantly depending on the used hydrogen-bond donors and acceptors in DES, as well as the properties of the plant material but they most often range from 1:10 to 1:40 [18]. The 1:50 SSR, which was optimal in the present study, also resulted in the highest yield of onion peel polyphenols when different DESs were used for the extraction in ratios ranging from 1:10 to 1:60 [22]. These results together suggest that at 1:60, saturation of the DES occurs, likely due to the maximization of the contact area and diffusion rates. In the case of 30% ethanol, the highest values of all tested parameters were observed at the ratio 1:60, which agrees with previous studies where the higher SSR within the applied ranges when using aqueous ethanol during MAE resulted in the highest yields of polyphenols [23,24].

MAE offers significantly shorter extraction times compared to conventional techniques, typically ranging from a few minutes to half an hour, to prevent oxidation and thermal degradation [25]. The dielectric properties of solvents influence how quickly they heat up, with longer exposure potentially degrading thermolabile compounds. Increased irradiation time may enhance recovery yields, while excessive durations may lead to diminished returns due to compound degradation [19]. Therefore, optimizing irradiation time is crucial in MAE to balance efficiency and compound integrity. In the present study, the extraction time significantly influenced the tested parameters for both DES and 30% ethanol. For DES, maximum values were achieved after 10 min, beyond which no further changes were detected. In contrast, 30% ethanol required the longest extraction time of 15 min to achieve the highest values. These differences are likely a result of the distinct dielectric properties of the solvents, which significantly influenced the extraction dynamics. This agrees with previous findings that have demonstrated that integrating advanced extraction methods like MAE with DES, rather than ethanol, water, or their blends, led to more effective extraction of polyphenols while concurrently shortening the extraction duration [26].

Temperature significantly impacts MAE by affecting the desorption rate, solubility, and degradation of targeted compounds. Typically, higher temperatures lead to increased extraction yields due to improved solvent diffusion into the plant matrix and enhanced solubility and desorption of the compounds from the matrix. However, applying higher temperatures can also result in the degradation of heat-sensitive compounds [27]. In the present study, the temperature had a significant effect on the content of polyphenols and antioxidant activity. Generally, higher values were achieved using DES compared to 30% ethanol, and the observed trends regarding temperature differed between the two solvents. When applying DES, an increase of the concentration of HCA and flavonoids was observed up to 60 °C, while at 80 °C a drop was observed. A similar phenomenon was observed for antioxidant activity; however, at 80 °C, there was a stagnation or, in the case of ABTS, a growth of antioxidant activity, which might be a result of the presence of some other antioxidants from banana peel extract, as well as the difference in mechanisms of the antioxidant activity assays. Unlike using DES, when 30% ethanol was applied as a solvent, the highest content of phenolic compounds and antioxidant activity was achieved at 80 °C. These differences between the solvents might be a result of decreased polarity of the DES with increasing temperature since it was shown that the increase of temperature is followed by a decrease of dielectric constant and relative polarity when observing the combination of choline chloride and glycerol with water as a cosolvent [28]. This effect might have been less pronounced when using 30% ethanol, as the polarity of ethanol decreases significantly when approaching subcritical conditions [29], which was not the case in the present study. Since polar solvents are generally more efficient at absorbing microwave energy due to their dipole moments and thus heat more rapidly and result in enhanced extraction procedure [19], the mentioned effects of temperature on polarity likely influenced the phenolic content and antioxidant activity in the present study.

Based on the results of statistical analysis, it can be concluded that the most favorable extraction conditions depended on the applied solvent. In the case of DES, the most favorable conditions were SSR 1:50, temperature 60 °C and extraction time 10 min, which represents a more energy- and solvent-efficient process compared to the SSR of 1:60, temperature 80 °C and 15 min, which were the most favorable when applying 30% ethanol. In addition, the content of HCA and flavonoids, as well as antioxidant activity, were notably higher in the extracts obtained by DES, thus adding to the benefits of choosing this solvent for MAE of banana peel polyphenols.

### 2.2. Optimization of UAE

In the present study, the influence of SSR, extraction time, and amplitude of the ultrasonic waves on the content of HCA and flavonoids, as well as the antioxidant activity determined by FRAP and ABTS methods, was examined and the results of statistical analysis are shown in Table 2.

As can be seen, all examined parameters had a statistically significant influence on HCA, flavonoids, and antioxidant activity. Generally, the SSR in the UAE has a significant impact on the efficiency of the process. A higher solvent ratio can improve solubilization, cavitation, and mass transfer but can result in a diluted extract and higher solvent costs. A lower ratio may be more economical and result in a more concentrated extract but may reduce extraction efficiency due to rapid solvent saturation and less efficient cavitation [30,31]. Therefore, the optimal ratio depends on the type of sample and the target compounds. In this study, in both cases, when DES and 30% ethanol were used as solvents, the increase in SSR was accompanied by an increase in HCA and flavonoid content and antioxidant activity. A larger solvent volume enables better mixing and a larger contact surface between the solvent and the sample. In addition, the ultrasonic waves generate cavitation bubbles in the solvent, which implode and cause microstructural changes in the sample. The larger the volume, the more cavitation bubbles are generated, which improves the degradation of cell structures and the release of polyphenols [30,32]. These findings agree with previous studies [5,33], where an increase in the SSR during UAE resulted in a higher yield of polyphenols and antioxidant activity.

Proper control of UAE time can significantly improve the yield and quality of the extract, while excessive or insufficient time can reduce the efficiency and economy of the process [34]. At the beginning of the ultrasonic extraction process, the cavitation effects of ultrasound cause swelling, hydration, and pore formation in the cell walls of the material from which the bioactive component is extracted. This accelerates the penetration of the solvent into the pores and enables the release of the bioactive substances into the solvent. However, prolonged exposure to ultrasound can lead to structural damage due to excessive heating, resulting in reduced extraction yield [32]. There may also be a decrease in polyphenol concentration, which may be the result of equilibrium conditions and the decomposition or transformation of the compound. In addition, continuous application of ultrasound may lead to collapse or compression of the plant matrix, reducing porosity and preventing further release of HCA. The subsequent increase could be due to further breakdown of the matrix and exposure of additional HCAs, along with possible solubilization of degradation products or changes in ultrasound wave patterns that can create areas of higher energy concentration at different times, possibly occasionally increasing extraction efficiency [30]. For this reason, process optimization is required. Usually, the optimal time for extraction of polyphenols from fruit and vegetable processing by-products with an ultrasound bath is between 10 and 90 min, and if an ultrasonic probe is used, it is even shorter [32,35]. In this study, when DES was used as a solvent, a shorter extraction time of 5 min was more efficient for better isolation of HCA and higher antioxidant activity of banana peel extracts, while a slightly longer time of 10 min was sufficient for the flavonoid content. This is likely because flavonoids are phenolic compounds with a complex structure that can contain multiple hydroxyl groups and different ring systems and can have stronger interactions with the plant matrix, making them more difficult to solubilize in DES, whereas HCA is smaller molecules with a simpler structure, making them easier to extract [36,37]. In the case of 30% ethanol, a longer extraction time was required to obtain higher values compared to DES. The reason for this could be the synergistic effect of ultrasound and DES, as ultrasonic waves in combination with a eutectic solvent can generate more efficient cavitation, which increases the intensity of microturbulence and the mechanical effect of ultrasound so that the extraction of polyphenols takes less time, which other studies confirmed [38,39]. As the mixture of choline chloride and glycerol also has excellent solubility properties, the polyphenols are more easily transferred from the sample into the solvent, which shortens the time required for extraction [40].

Amplitude plays a key role in ultrasound extraction, as it has a direct effect on the intensity of the ultrasonic waves and, therefore, on the extraction efficiency. Proper control of amplitude allows the extraction process to be optimized by striking a balance between maximum extraction efficiency and preservation of extract quality. Too high an amplitude can lead to undesirable effects, such as the thermal degradation of compounds, while too low an amplitude can result in inefficient extraction [41]. The highest values for HCA, ABTS, and FRAP were obtained at 75% amplitude, after which a decrease was observed when DES was used as the solvent, while the highest flavonoid content was obtained at 50% amplitude. When 30% ethanol was used, an increase in HCA and flavonoid content and FRAP values was observed up to 100% amplitude, while the highest ABTS values were obtained at 75% amplitude. The reason for achieving higher antioxidant activity with the ABTS method at low amplitude is likely due to the difference in the reaction mechanism compared to the FRAP method. In addition, lower amplitude values were required for efficient extraction when DES was used as solvent compared to 30% ethanol. As mentioned above, DES has a better ability to dissolve polyphenols due to its intermolecular interactions, creates more efficient cavitation and energy transfer, and has a lower viscosity, which reduces the need for high amplitudes as polyphenols move faster from the sample to the solvent [30].

In general, the optimal conditions for efficient extraction of phenolic compounds and high antioxidant activity were SSR 1:60, 5 min extraction time, and 75% amplitude for DES and SSR 1:60, 15 min extraction time, and 100% amplitude for 30% ethanol. When comparing the results obtained with DES and 30% ethanol, it can be seen that the contents obtained with DES were up to 7-fold higher in some cases, indicating that it is the better solvent for the extraction of phenolic compounds from banana peel.

### 2.3. Phenolic Profile of Banana Peel Extracts Obtained at Optimal Conditions

To investigate the phenolic profile of the banana peel extracts obtained at defined optimal extraction parameters, UPLC-MS^2^ analysis was carried out. In all extracts, 19 phenolic compounds belonging to the groups of flavonols, flavan-3-ols, and phenolic acids were detected (Table 3).

Among flavonols, compounds **8**, **10**, **12**, **14**, and **19** were identified through comparison with authentic standards such as myricetin, quercetin-3-glucoside, kaempferol-3-glucoside, rutin, and quercetin-3-galactoside. Compounds **11** and **15** were assigned as quercetin-3-glucuronide and kaempferol-3-glucuronide according to their fragmentation patterns, as reported previously [42]. The main flavonol representatives were myricetin and rutin whose concentration varied depending on the applied solvent and extraction technique. These two compounds were also found to be the most abundant flavonols in 95% ethanolic banana peel extract [43], as well as in methanolic banana peel extract [44], where rutin was more prevalent than myricetin. In another study, kaempferol and quercetin glycosides were the main flavonols found in banana peel extract incorporated into functional yogurt [45]. As for flavan-3-ols, compounds **3** and **7** were identified through comparison with authentic standards as catechin and epicatechin gallate, while compound **9** was assigned as epicatechin, as reported previously [46]. All three of these compounds were previously found to be present in banana peel in relevant concentrations, with epicatechin being dominant [43,45,47]. Among phenolic acids, compounds **1**, **4**, **5**, **6**, and **16** were identified by comparison to the reference standard curve as neochlorogenic, caffeic, *p*-coumaric, chlorogenic, and gallic acid, respectively. Compounds **2**, **13**, **17**, and **18** were identified through their fragmentation patterns described previously as 3,4-dihydrobenzoic acid hexoside [46], ellagic acid [48], *p*-hydroxybenzoic acid [46], and caftaric acid [49], respectively. Chlorogenic, neochlorogenic, and caffeic acid were the main representatives of phenolic acids, which is partially in agreement with results obtained by Anwar et al. [45], where chlorogenic acid was the most abundant and caffeic acid was present in high amounts. In that research, however, neochlorogenic acid was not identified. In a study by Athanasiadis et al. [1], rosmarinic acid, which was not detected in our extract, was the most abundant phenolic acid, followed by chlorogenic and neochlorogenic acid. On the other hand, gallic and ellagic acids were the most abundant phenolic acids in a methanolic banana peel extract [44], while in the present research, the concentration of these acids was relatively low. The differences are likely a result of different banana varieties, as well as the applied extraction techniques and used solvents. The effect of solvent is visible in the results of the present study, as well.

A higher total polyphenols content was extracted using DESs in both techniques and differences in the proportions of various phenolic groups were observed between the extraction techniques. UAE resulted in a higher content of total polyphenols than MAE when applying DES, while MAE resulted in a higher content of total polyphenols than UAE when applying 30% ethanol. This is likely a result of combined effects, but especially the effect of temperature on solvents during MAE, which was explained in Section 2.1. That resulted in lower extraction efficiency compared to UAE, which was carried out by maintaining the temperature below 37 °C. MAE was more efficient when using 30% ethanol, possibly also due to the effect of temperature, which was 80 °C in the optimal extract, which allowed improved solvent diffusion into the plant matrix and enhanced solubility and desorption of the compounds from the matrix [27]. With the application of DES, flavan-3-ols were the most abundant in both extraction techniques, which is likely a result of strong interactions of the DES and hydroxyl and carbonyl groups of the flavan-3-ols through hydrogen bonding [36]. When using 30% ethanol in both extraction techniques, however, more flavonols (namely myricetin) than flavan-3-ols were extracted. Since myricetin, as the main representative of flavonols, contains an extra carbonyl and hydroxyl group in its structure compared to catechin and epicatechin [50], it would be expected to obtain a higher yield in both 30% ethanol and DES due to higher potential for hydrogen bonding [51,52]. However, it is possible that the activation energy of myricetin required for desorption from the matrix was too high, and the conditions used for extraction with 30% ethanol (longer extraction time, higher temperature (MAE), and amplitude (UAE)) were more favorable for achieving its higher yields. A higher concentration of phenolic acids was obtained using DES in both techniques, namely due to the content of neochlorogenic and chlorogenic acids, which possess more functional groups than the caffeic acid [53] that was the most abundant in 30% ethanol, which likely allowed more favorable interactions between the DES components and the phenolic acids. In support, DES comprised of choline chloride and poly-alcohols such as glycerol were shown to be highly successful compared to other solvents for extraction of neochlorogenic and chlorogenic acid [54]. In 30% ethanol, caffeic acid was the most abundant, while the content of neochlorogenic acid and chlorogenic acid was significantly lower. It is likely that caffeic acid was more successfully extracted in 30% ethanol due to its simple, non-esterified structure (higher polarity) and lower molecular mass [53], as well as the susceptibility of neochlorogenic and chlorogenic to thermal and ultrasonic degradation [53,55] since the temperature and amplitude when using 30% ethanol were higher than when using DESs.

The content of both flavonoids (flavonols and flavan-3-ols combined) and hydroxycinnamic acids (HCA) was significantly lower than the one detected by the spectrophotometric analysis, likely due to the overestimation by spectrophotometric techniques as a result of overlapping absorption bands, interference from other compounds that absorb at similar wavelengths, matrix effects, the presence of precursors or degradation products that still absorb at the same wavelength, reagent-specific reactions with other phenolic compounds or reducing substances, and the influence of pH and solvent effects on absorbance properties [56,57]. Nevertheless, the results of UPLC-MS^2^ analysis confirmed the trends in quantities of flavonoids and hydroxycinnamic acids as affected by the type of solvent that was observed in the results of spectrophotometric analyses, thus confirming that these methods can be used for estimating the influence of extraction parameters on the content of polyphenolic groups in the extract but emphasizing the need for the use of chromatographic techniques for precise results.

## 3. Materials and Methods

### 3.1. Chemicals and Reagents

Purified water was prepared utilizing the advanced Milli-Q system (Millipore, Bedford, NY, USA). High-performance liquid chromatography (HPLC) grade acetonitrile and acetic acid (99%) were procured from J.T. Baker Chemicals in Deventer, The Netherlands. The Folin–Ciocalteu reagent was acquired from Merck (Darmstadt, Germany). Ethanol (p.a.) was sourced from Kemika d.d. (Zagreb, Croatia). Iron (III) chloride hexahydrate, sodium acetate trihydrate, and sodium carbonate were obtained from Kemika d.d. (Zagreb, Croatia). Trolox and 2,4,6-tri(2-pyridyl)-s-triazine (TPTZ) were procured from Acros Organics (Geel, Belgium). High-purity methanol (99%) was sourced from Honeywell (Riedel-de-Haën, Bucharest, Romania). 2,2-Diphenyl-1-picrylhydrazyl (DPPH) was acquired from Sigma–Aldrich (St. Louis, MO, USA). Hydrochloric acid (HCl, 37%) was sourced from Carlo Erba Reagents (Val-de-Reuil, France). Choline chloride (99.5%) and glycerol were procured from Biovit d.o.o. (Jalkovec, Croatia). HPLC standards of rutin, quercetin-3-galactoside, quercetin-3-glucoside, kaempferol-3-glucoside, myricetin, epicatechin gallate, catechin, chlorogenic, neochlorogenic, caffeic, gallic and *p*-coumaric acid were procured from Sigma–Aldrich (St. Louis, MO, USA). Standard stock solutions were produced in methanol and used for the preparation of working standard solutions (10–200 mg/L) by diluting the prepared stock solutions, ensuring precise calibration.

### 3.2. Sample Preparation

The commercially available Cavendish banana (*Musa acuminata*) samples were purchased at a local market in Zagreb, Croatia, and the peels were manually separated and freeze-dried for 48 h at −55 °C (Alpha 1–4 LSCPlus, Osterode am Harz, Germany). The dried banana peels were ground with an electric grinder and stored at −18 °C until extraction.

### 3.3. Deep Eutectic Solvent Preparation

The weighed masses of the substances calculated according to the ratios specified in the experimental design (Supplementary Materials; Table S1) were mixed in a laboratory beaker, a magnet was added to the beaker, and it was placed on a magnetic stirrer at a temperature of 50 °C, and mixing was carried out until a homogeneous, clear liquid was obtained.

### 3.4. Extraction

The experimental design for the MAE and UAE studies is shown in Table 4. In the MAE, the parameters varied were the SSR, time and temperature, while in the UAE, the amplitude was varied as a parameter instead of temperature. Two types of solvents were used in both extractions: DES with choline chloride and glycerol in a 1:3 ratio with a water content of 30% (*w*/*w*) and 30% ethanol (*v*/*v*). DES was selected based on preliminary studies (Supplementary Materials; Table S1), with choline chloride as a hydrogen-bond acceptor and lactic acid, glycerol, and fructose as hydrogen-bond donors. The molar ratio was constant, while the water content in DES varied between 10 and 30%. The selected DES showed the highest proportions of HCA and flavonoids. The 30% ethanol was chosen for its GRAS status and as an excellent solvent for efficient extraction of phenolic compounds [58,59].

#### 3.4.1. MAE

The MAE of phenolic compounds from freeze-dried banana peels was performed in the Ethos Easy Reactor (Milestone, Sorisole, Italy) by weighing the required weight of crushed sample into the extraction cell according to the indicated SSR and adding 40 mL of extraction solvent and a magnetic stirrer. The extraction cell with the mixture was placed in the reactor, and the extraction was carried out according to the experimental design with constant parameters: microwave power 400 W, stirring power 50%, heating time to the desired temperature 2 min for 40 °C, 4 min for 60 °C, 6 min for 80 °C, ventilation and cooling after extraction for 2 min, and the temperature was kept constant during the extraction process. After completion of the extraction, the mixture was filtered into a 50 mL volumetric flask, topped up to the mark with solvent, and stored at −18 °C until analyzed.

#### 3.4.2. UAE

The UAE of phenolic compounds from freeze-dried banana peels was performed by weighing the required amount of crushed sample into a beaker according to the indicated SSR and adding 40 mL of the extraction solvent. The mixture was stirred, and the extraction was carried out with an ultrasonic probe UP200Ht (Dr Hielscher GmbH, Teltow, Germany) according to the experimental plan (Table 4) at a temperature of ≤30 °C in a cold bath. The constant parameters were an output power of 200 W and an ultrasonic frequency of 26 kHz. After completion of the extraction, the mixture was filtered into a 50 mL volumetric flask, filled up to the mark with the solvent, and stored at −18 °C until analyzed.

### 3.5. Spectrophotometric Determination of Total Flavonoids and Hydroxycinnamic Acids

#### 3.5.1. Total Flavonoids

To measure the total flavonoids, 0.5 mL of the extract, 1.5 mL of 96% ethanol, 0.1 mL of 10% aluminum chloride, 0.1 mL of 1 M potassium acetate, and 2.8 mL of distilled water were combined in a glass tube. A blank sample was prepared similarly, using the extraction solvent instead of the extract and 0.1 mL of distilled water instead of aluminum chloride. The reaction mixture was left to stand for 30 min before measuring the absorbance at 415 nm. A calibration curve was established using quercetin standard solutions ranging from 10 to 100 mg/L. Results were expressed in mg/100 g of dry banana peel.

#### 3.5.2. HCA

In summary, 250 μL of the extract, 250 μL of 1 g/L HCl in 96% ethanol, and 4.55 mL of 2 g/L HCl were combined in a glass tube. A blank sample was prepared similarly, using the extraction solvent in place of the extract. The total HCA was quantified by measuring the absorbance at 320 nm. A calibration curve for chlorogenic acid was created using standard solutions with concentrations from 10 to 100 mg/L. The results were expressed in mg/100 g of dry banana peel.

### 3.6. Antioxidant Activity Assays

#### 3.6.1. Ferric Reducing Antioxidant Power Assay (FRAP)

The FRAP assay was performed according to the method of Benzie and Strain [60] and Dabetić et al. [11] with some modifications. To measure the absorbance of the samples to determine the antioxidant capacity, 240 µL of distilled water, 80 µL of the sample, and 2080 µL of the FRAP reagent were pipetted into the test tubes. The prepared samples were mixed with a vortex mixer and thermostatted for 30 min at 37 °C in the dark. Everything was added to the blank except the sample in place of which the extraction solvent was taken. The absorbance was measured at 593 nm. A calibration curve for Trolox was constructed using working standard solutions, and the results were expressed as mmol of Trolox equivalents (TE)/100 g of dry banana peel.

#### 3.6.2. 2,2-Azinobis(3-Ethylbenzothiazoline-6-Sulfonic Acid) Assay (ABTS)

The ABTS assay was carried out according to the method described by Miller and Rice-Evans [61] with some modifications. Briefly, 160 μL of the diluted sample was mixed with 2 mL of 1% ABTS•+, and after 1 min, the absorbance was measured at 734 nm. For the blank test, 96% ethanol was used. A calibration curve for Trolox was created using working standard solutions, and results were expressed in mmol TE/100 g of dry banana peel.

### 3.7. Determination of the Extracts’ Phenolic Profile

The banana peel extracts obtained through MAE and UAE using DES and 30% ethanol under optimal conditions were filtered into glass vials using 0.45 μm syringe filters. Subsequently, they underwent phenolic profile analysis via UPLC-MS^2^ utilizing the Agilent series 1290 RRLC instrument paired with an Agilent 6430 Triple Quad LC/MS mass spectrometer alongside Agilent MassHunter Workstation Software (ver. B.5.0.591.0) for data processing and instrument control. Ionization of analytes occurred through the ESI ion source in both positive and negative modes. Nitrogen was employed for desolvation and collision at a flow rate of 11 L/h, with a temperature of 300 °C, nebulizer pressure set at 2.76 bar, and a capillary voltage of 4/−3.5 kV. Separation was facilitated by a C18 column (Agilent Zorbax Eclipse Plus; dimensions: 100 × 2.1 mm; particle size: 1.8 µm) maintained at 35 °C, with an injection volume of 2.5 µL. Details regarding solvent composition, gradient conditions, collision energy for individual compounds, and analytical quality parameters, such as detection (LOD) and quantification limits (LOQ), were previously outlined [62]. Compound identification and quantification relied on the standard calibration curves or, in the absence of reference standards, comparison of mass fragmentation patterns with existing literature reports. Quantification of compounds lacking reference standards was performed as follows: quercetin-3-glucuronide according to quercetin-3-glucoside, caftaric acid according to caffeic acid, ellagic and *p*-hydroxybenzoic acid according to gallic acid and 3,4-dihydrobenzoic acid hexoside according to protocatechuic acid. Concentrations of identified polyphenols were expressed as mg/100 g of dry banana peel.

### 3.8. Statistical Analysis

Statistical analysis was conducted utilizing Statistica version 10.0 software (Statsoft Inc., Tulsa, OK, USA). Each extraction and analysis was performed in duplicate. A mixed-level full-factorial experimental design consisting of 27 trials was used to assess the effects of three independent variables in both UAE and MAE. In UAE, the independent variables were SSR, time, and amplitude, while in MAE, it was temperature instead of amplitude, and the dependent variables in both cases were HCA, flavonoids, ABTS, and FRAP of banana peel extracts. The obtained data and residuals were tested for normality and homoscedasticity using the Shapiro–Wilk test and Levene’s test, respectively. Based on these assessments, the data were analyzed using ANOVA followed by Tukey’s HSD post-hoc test or the non-parametric Kruskal–Wallis test with multiple comparisons of mean ranks. Differences in phenolic composition between the optimized MAE and UAE extracts were assessed by one-way ANOVA followed by Tukey’s HSD post-hoc test. The significance level for all tests was set at *p* ≤ 0.05.

## 4. Conclusions

In this study, for the first time, the parameters of two advanced extraction techniques, MAE and UAE, were optimized to isolate HCA and flavonoids from banana peels and to determine the antioxidant activity of the extracts obtained using DES consisting of choline chloride and glycerol, and using 30% ethanol for comparison. The results showed that banana peels are a rich source of polyphenols with high antioxidant properties. In MAE, the optimal conditions for efficient polyphenol isolation and high antioxidant activity with DES were an SSR of 1:50, a temperature of 60 °C, and an extraction time of 10 min. With 30% ethanol, the optimal conditions were an SSR of 1:60, a temperature of 80 °C, and an extraction time of 15 min. For UAE, the optimal conditions were an SSR of 1:60, an extraction time of 5 min, and a 75% amplitude for DES, and an SSR of 1:60, an extraction time of 15 min, and a 100% amplitude for 30% ethanol. The phenolic profile of the optimal extracts included 19 compounds belonging to the groups of flavonols, flavan-3-ols, and phenolic acids. In the extracts with DES, flavan-3-ols, especially catechin and epicatechin, were the most abundant. In the extracts with 30% ethanol, flavonols were the most abundant, with myricetin as the most valuable component. Both techniques tested proved to be effective for the isolation of antioxidant phenolic compounds. The study concluded that choline chloride and glycerol in a 1:3 ratio with a water content of 30% is a highly efficient and environmentally friendly solvent for the extraction of valuable bioactive compounds from banana peels, which is promising for applications in the food and pharmaceutical industries.

## Figures and Tables

**Table 1:** The influence of MAE parameters on the content of HCA, flavonoids and the antioxidant activity determined by FRAP and ABTS methods.

Variation	HCA (mg/100 g)		Flavonoids (mg/100 g)		FRAP (mmol/100 g)		ABTS (mmol/100 g)	
	DES	30% EtOH	DES	30% EtOH	DES	30% EtOH	DES	30% EtOH
SSR (g/mL)	*p* < 0.01 *	*p* < 0.01 *	*p* < 0.01 *	*p* < 0.01 *	*p* < 0.01 *	*p* < 0.01 *	*p* < 0.01 *	*p* < 0.01 *
1:40	274.82 ± 12.91 ^a^	84.87 ± 3.26 ^a^	136.27 ± 5.38 ^a^	27.19 ± 1.44 ^a^	39.19 ± 1.49 ^c^	4.98 ± 0.45 ^a^	14.02 ± 0.85 ^a^	4.82 ± 0.20 ^a^
1:50	313.52 ± 12.68 ^b^	116.10 ± 5.42 ^b^	145.50 ± 5.52 ^b^	36.41 ± 2.93 ^b^	32.58 ± 1.32 ^b^	5.91 ± 0.50 ^b^	15.55 ± 0.86 ^b^	6.30 ± 0.26 ^b^
1:60	324.90 ± 10.33 ^b^	127.60 ± 6.45 ^c^	154.66 ± 4.75 ^b^	43.30 ± 2.84 ^c^	29.18 ± 2.74 ^a^	8.77 ± 0.87 ^c^	18.64 ± 1.35 ^c^	6.84 ± 0.44 ^c^
Time (min)	*p* < 0.01 *	*p* < 0.01 *	*p* < 0.01 *	*p* < 0.01 *	*p* < 0.01 *	*p* < 0.01 *	*p* < 0.01 *	*p* < 0.01 *
5	286.78 ± 12.80 ^a^	103.21 ± 6.81 ^a^	134.27 ± 6.30 ^a^	33.93 ± 2.94 ^a^	31.94 ± 1.63 ^a^	6.05 ± 0.86 ^a^	15.62 ± 1.15 ^a^	5.54 ± 0.40 ^a^
10	313.02 ± 13.13 ^b^	101.10 ± 4.64 ^a^	152.42 ± 3.77 ^b^	33.15 ± 1.87 ^a^	34.70 ± 2.74 ^b^	6.72 ± 0.65 ^b^	15.67 ± 1.13 ^a^	6.08 ± 0.36 ^b^
15	313.45 ± 12.32 ^b^	124.26 ± 7.20 ^b^	149.74 ± 5.18 ^b^	39.82 ± 3.56 ^b^	34.32 ± 2.03 ^b^	6.89 ± 0.70 ^c^	16.92 ± 0.12 ^b^	6.33 ± 0.36 ^c^
Temperature (°C)	*p* < 0.01 *	*p* < 0.01 *	*p* < 0.01 *	*p* < 0.01 *	*p* < 0.01 *	*p* < 0.01 *	*p* < 0.01 *	*p* < 0.01 *
40	320.17 ± 7.69 ^b^	108.52 ± 4.28 ^b^	137.30 ± 3.38 ^a^	34.36 ± 1.04 ^b^	30.50 ± 2.74 ^a^	4.96 ± 0.16 ^a^	12.63 ± 0.30 ^a^	5.23 ± 0.16 ^a^
60	341.01 ± 8.61 ^c^	102.28 ± 6.76 ^a^	161.75 ± 4.42 ^b^	31.72 ± 3.59 ^a^	32.86 ± 1.72 ^b^	6.22 ± 0.78 ^b^	13.90 ± 0.56 ^b^	5.45 ± 0.34 ^b^
80	252.05 ± 11.29 ^a^	117.78 ± 8.24 ^c^	137.38 ± 6.13 ^a^	40.82 ± 3.14 ^c^	36.60 ± 2.58 ^b^	8.48 ± 0.81 ^c^	21.68 ± 0.83 ^c^	7.27 ± 0.38 ^c^
mean	304.41	109.52	145.48	35.63	33.65	6.55	16.07	5.98

SSR = sample:solvent ratio. DES = deep eutectic solvent. EtOH = ethanol. HCA = hydroxycinnamic acids. * Statistically significant variable at *p* ≤ 0.05. Values are expressed as mean ± SD. Values within cells marked with different letters are statistically different at *p* ≤ 0.05.

**Table 2:** The influence of UAE parameters on the content of HCA, flavonoids and the antioxidant activity determined by FRAP and ABTS methods.

Variation	HCA (mg/100 g)		Flavonoids (mg/100 g)		FRAP (mmol/100 g)		ABTS (mmol/100 g)	
	DES	30% EtOH	DES	30% EtOH	DES	30% EtOH	DES	30% EtOH
SSR (g/mL)	*p* < 0.01 *	*p* < 0.01 *	*p* < 0.01 *	*p* < 0.01 *	*p* < 0.01 *	*p* < 0.01 *	*p* < 0.01 *	*p* < 0.01 *
1:40	322.29 ± 19.84 ^a^	113.11 ± 4.28 ^a^	206.59 ± 16.33 ^a^	49.19 ± 2.29 ^a^	27.79 ± 2.21 ^a^	4.08 ± 0.27 ^a^	19.49 ± 0.91 ^a^	3.84 ± 0.37 ^a^
1:50	483.04 ± 28.10 ^b^	139.44 ± 3.79 ^b^	332.21 ± 17.67 ^b^	51.84 ± 2.13 ^b^	30.04 ± 2.38 ^b^	5.27 ± 0.19 ^b^	21.99 ± 1.51 ^b^	6.03 ± 0.18 ^b^
1:60	540.76 ± 11.51 ^c^	167.52 ± 9.09 ^c^	410.33 ± 19.55 ^c^	74.03 ± 6.96 ^c^	37.15 ± 3.44 ^c^	5.93 ± 0.34 ^c^	22.59 ± 1.38 ^c^	6.46 ± 0.25 ^c^
Time (min)	*p* < 0.01 *	*p* < 0.01 *	*p* < 0.01 *	*p* < 0.01 *	*p* < 0.01 *	*p* < 0.01 *	*p* < 0.01 *	*p* < 0.01 *
5	461.75 ± 20.19 ^b^	129.52 ± 5.47 ^a^	299.38 ± 26.40 ^a^	50.59 ± 2.81 ^a^	33.23 ± 2.67 ^b^	4.94 ± 0.28 ^a^	23.35 ± 1.42 ^c^	4.80 ± 0.53 ^a^
10	425.45 ± 29.27 ^a^	137.88 ± 5.50 ^b^	342.45 ± 25.31 ^b^	55.50 ± 2.29 ^b^	30.10 ± 1.92 ^a^	5.00 ± 0.24 ^a^	21.21 ± 1.19 ^b^	5.78 ± 0.21 ^b^
15	458.90 ± 38.88 ^b^	152.67 ± 11.23 ^c^	307.30 ± 28.37 ^a^	68.97 ± 7.51 ^c^	31.65 ± 4.29 ^ab^	5.35 ± 0.43 ^b^	19.51 ± 1.21 ^a^	5.74 ± 0.33 ^b^
Amplitude (%)	*p* < 0.01 *	*p* < 0.01 *	*p* < 0.01 *	*p* < 0.01 *	*p* < 0.01 *	*p* < 0.01 *	*p* < 0.01 *	*p* < 0.01 *
50	413.99 ± 26.81 ^a^	131.66 ± 4.64 ^a^	368.03 ± 19.23 ^c^	52.40 ± 2.18 ^a^	26.95 ± 2.19 ^a^	4.04 ± 0.26 ^a^	17.69 ± 0.94 ^a^	5.04 ± 0.24 ^a^
75	495.13 ± 36.93 ^c^	141.71 ± 5.57 ^b^	318.34 ± 36.31 ^b^	57.43 ± 3.60 ^b^	34.27 ± 3.35 ^b^	5.49 ± 0.24 ^b^	21.51 ± 1.05 ^b^	5.78 ± 0.43 ^c^
100	436.97 ± 23.14 ^b^	146.70 ± 11.95 ^c^	262.77 ± 13.80 ^a^	65.23 ± 7.58 ^c^	33.75 ± 2.68 ^b^	5.75 ± 0.33 ^c^	24.88 ± 1.35 ^b^	5.51 ± 0.45 ^b^
mean	448.70	140.02	316.38	58.35	31.66	5.09	21.36	5.44

SSR = sample:solvent ratio. DES = deep eutectic solvent. EtOH = ethanol. HCA = hydroxycinnamic acids. * Statistically significant variable at *p* ≤ 0.05. Values are expressed as mean ± SD. Values within cells marked with different letters are statistically different at *p* ≤ 0.05.

**Table 3:** Phenolic profile of the banana peel extracts obtained by MAE and UAE at the optimal extraction conditions with DES and 30% ethanol.

Compound Number	Retention Time (min)	Precursor Ion (*m*/*z*)	Fragment Ion (*m*/*z*)	Tentative Identification	Concentration mg/100 g Banana Peel			
					MAE		UAE	
					DES	30% EtOH	DES	30% EtOH
Flavonols								
8	6.298	319	273	Myricetin *	2.32 ± 0.07 ^aA^	17.06 ± 0.48 ^bB^	6.25 ± 0.18 ^aB^	11.80 ± 0.33 ^bA^
10	7.469	465	303	Quercetin-3-glucoside *	1.46 ± 0.04 ^bB^	0.63 ± 0.02 ^aB^	1.34 ± 0.04 ^bA^	0.35 ± 0.01 ^aA^
11	7.474	479	303	Quercetin-3-glucuronide	0.37 ± 0.01 ^aA^	0.63 ± 0.02 ^bB^	0.35 ± 0.01 ^bA^	0.10 ± 0.00 ^aA^
12	7.477	449	287	Kaempferol-3-glucoside *	0.70 ± 0.02 ^bB^	0.39 ± 0.01 ^aB^	0.64 ± 0.02 ^bA^	0.24 ± 0.01 ^aA^
14	9.456	611	465/303	Rutin *	7.40 ± 0.21 ^aA^	7.01 ± 0.20 ^aB^	7.03 ± 0.20 ^bA^	0.86 ± 0.02 ^aA^
15	11.389	463	287	Kaempferol-3-glucuronide	0.16 ± 0.00 ^aA^	0.18 ± 0.00 ^aA^	0.28 ± 0.01 ^aB^	0.26 ± 0.01 ^aB^
19	12.211	465	303	Quercetin-3-galactoside *	1.58 ± 0.04 ^bA^	0.92 ± 0.03 ^aA^	1.62 ± 0.05 ^bA^	1.38 ± 0.04 ^aB^
				**Total flavonols**	13.99 ± 0.40 ^aA^	26.82 ± 0.76 ^bB^	17.52 ± 0.50 ^bB^	15.00 ± 0.42 ^aA^
Flavan-3-ols								
3	3.825	291	165/139	Catechin *	16.29 ± 0.46 ^bA^	4.63 ± 0.13 ^aB^	18.99 ± 0.54 ^bB^	3.24 ± 0.09 ^aA^
7	6.235	442.9	273	Epicatechin gallate *	4.99 ± 0.14 ^aB^	7.78 ± 0.22 ^bB^	3.39 ± 0.10 ^aA^	3.03 ± 0.09 ^aA^
9	6.529	291	139/123	Epicatechin	11.61 ± 0.33 ^bA^	5.48 ± 0.16 ^aA^	19.02 ± 0.54 ^bB^	8.01 ± 0.23 ^aB^
				**Total flavan-3-ols**	32.89 ± 0.93 ^bA^	17.89 ± 0.51 ^aB^	41.44 ± 1.17 ^bB^	14.29 ± 0.93 ^aA^
Phenolic acids								
1	1.895	353	191/173	Neochlorogenic acid *	7.70 ± 0.22 ^bB^	0.92 ± 0.03 ^aB^	5.31 ± 0.15 ^bA^	0.76 ± 0.02 ^aA^
2	3.713	137	109	3,4-dihydrobenzoic acid hexoside	0.06 ± 0.00 ^aB^	0.13 ± 0.00 ^bB^	0.04 ± 0.00 ^aA^	0.07 ± 0.00 ^bA^
4	4.893	179	135	Caffeic acid *	2.47 ± 0.07 ^aB^	5.36 ± 0.15 ^bB^	0.29 ± 0.01 ^aA^	3.86 ± 0.11 ^bA^
5	5.492	163	119	*p*-coumaric acid *	0.30 ± 0.01 ^aB^	1.35 ± 0.04 ^bB^	0.24 ± 0.01 ^aA^	0.84 ± 0.02 ^bA^
6	5.561	353	191	Chlorogenic acid *	8.73 ± 0.25 ^bB^	0.68 ± 0.02 ^aB^	6.05 ± 0.17 ^bA^	0.40 ± 0.01 ^aA^
13	9.299	300.9	255.3/145	Ellagic acid	0.28 ± 0.01 ^aA^	0.28 ± 0.01 ^aA^	0.43 ± 0.01 ^bB^	0.27 ± 0.01 ^aA^
16	11.408	169	125	Gallic acid *	1.33 ± 0.04 ^bA^	0.48 ± 0.01 ^aA^	1.45 ± 0.04 ^bB^	0.53 ± 0.01 ^aB^
17	11.728	137	93	*p*-hydroxybenzoic acid	0.80 ± 0.02 ^bA^	0.67 ± 0.02 ^aB^	0.83 ± 0.02 ^bA^	0.61 ± 0.02 ^aA^
18	11.991	311.1	179/149	Caftaric acid	0.06 ± 0.00 ^bA^	0.04 ± 0.00 ^aA^	0.06 ± 0.00 ^bA^	0.05 ± 0.00 ^aB^
				**Total phenolic acids**	21.74 ± 0.61 ^bB^	9.92 ± 0.28 ^aB^	14.71 ± 0.42 ^bA^	7.39 ± 0.21 ^aA^
				**Hydroxycinnamic acids**	19.54 ± 0.55 ^bB^	8.64 ± 0.24 ^aB^	12.39 ± 0.35 ^bA^	6.18 ± 0.17 ^aA^
				**Total polyphenols**	68.62 ± 1.94 ^bA^	54.64 ± 1.55 ^aB^	73.64 ± 2.08 ^bB^	36.67 ± 1.08 ^aA^
				**Flavonols (%)**	20.39 ^aA^	49.09 ^bB^	23.79 ^aB^	40.90 ^bA^
				**Flavan-3-ols (%)**	47.93 ^bA^	32.75 ^aA^	56.23 ^bB^	38.95 ^aB^
				**Phenolic acids (%)**	31.68 ^bB^	18.16 ^aA^	19.98 ^aA^	20.15 ^aB^

* identification confirmed by comparison with an authentic standard. MAE = microwave-assisted extraction. UAE = ultrasound-assisted extraction. DES = deep eutectic solvent. EtOH = ethanol. Values are expressed as mean ± SD. Different lowercase letters attributed to values indicate a statistically significant difference within technique depending on the solvent, while different uppercase letters indicate a significant difference between two techniques within one solvent type at *p* ≤ 0.05.

**Table 4:** Experimental design.

Sample:Solvent (g/mL)	Time (min)	Temperature (°C) ^a^	Amplitude (%) ^b^
1:40	5	40	50
		60	75
		80	100
	10	40	50
		60	75
		80	100
	15	40	50
		60	75
		80	100
1:50	5	40	50
		60	75
		80	100
	10	40	50
		60	75
		80	100
	15	40	50
		60	75
		80	100
1:60	5	40	50
		60	75
		80	100
	10	40	50
		60	75
		80	100
	15	40	50
		60	75
		80	100

^a^ varied in MAE, ^b^ varied in UAE.

## Data Availability

The original contributions presented in the study are included in the article/Supplementary Materials. Further inquiries can be directed to the corresponding authors.

## Supplementary Materials

The following supporting information can be downloaded at: https://www.mdpi.com/article/10.3390/molecules29153672/s1, Figure S1: UPLC-MS2 chromatogram in MRM acquisition mode of banana peel extract obtained in DES by MAE; Table S1: Preliminary content of banana peel polyphenols in extracts obtained using different deep eutectic solvents; Table S2: Content of hydroxycinnamic acids, flavonoids and antioxidant activity of banana peel extracts obtained by MAE and UAE.
