# Supplementary material for: Green Approaches for the Extraction of Banana Peel Phenolics Using Deep Eutectic Solvents
Source: Molecules. 2024 Aug 2;29(15):3672. doi: 10.3390/molecules29153672 (PMC11314314; doi:10.3390/molecules29153672)
Supplement: Supplementary file 1 [file molecules-29-03672-s001.zip › Figure S1.pdf]

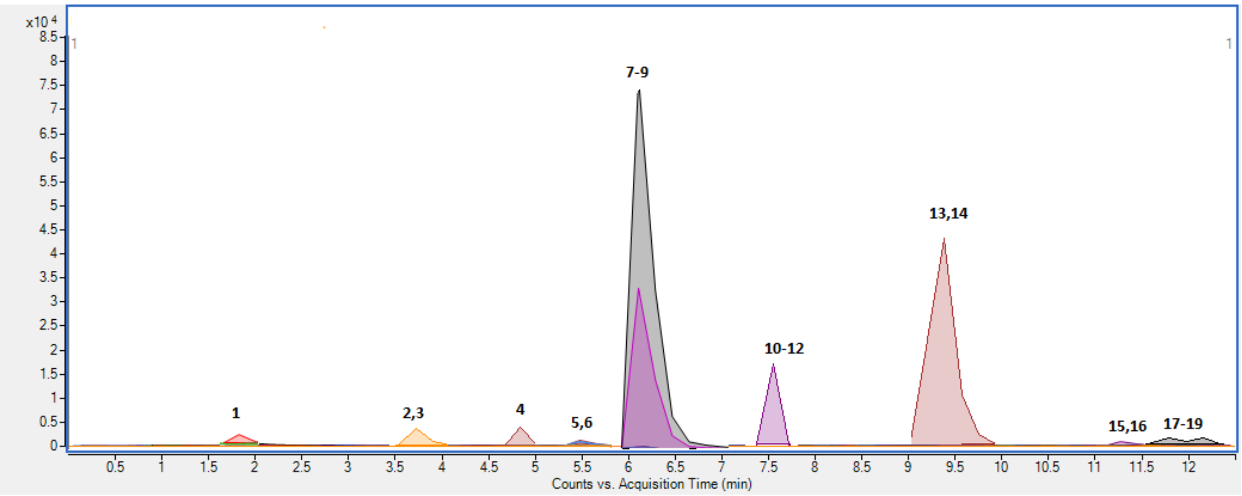

Figure S1. UPLC-MS² chromatogram in MRM acquisition mode of banana peel extract obtained in DES by MAE (1) neochlorogenic acid, (2) 3,4-dihhidroxybenzaldehyde, (3) catehin, (4) caffeic acid, (5) *p*-coumaric acid, (6) chlorogenic acid, (7) myricetin, (8) epicatechin gallate, (9) epicatehin, (10) quercetin -3-glucoside, (11) quercetin -3-glucuronide, (12) kaempferol-3-glucoside, (13) ellagic acid, (14) rutin, (15) kaempferol -3-glucuronide, (16) Gallic acid, (17) *p*-hydroxybenzoic acid, (18) caftaric acid, (19) quercetin-3-galactoside.
